# A National, Electronic Health Record–Based Study of Perinatal Hemorrhagic and Ischemic Stroke

**DOI:** 10.1177/08830738231170739

**Published:** 2023-04-25

**Authors:** Stuart Fraser, Samantha M. Levy, Yashar Talebi, Sean I. Savitz, Alicia Zha, Gen Zhu, Hulin Wu

**Affiliations:** 1 Division of Vascular Neurology, Department of Neurology, 12339 McGovern Medical School, University of Texas Health Science Center at Houston, Houston, TX, USA; 2 Institute for Stroke and Cerebrovascular Disease, University of Texas Health Science Center at Houston, Houston, TX, USA; 3 Department of Biostatistics and Data Science, 12340 School of Public Health, University of Texas Health Science Center at Houston, Houston, TX, USA; 4 Division of Vascular Neurology, Department of Neurology, Ohio State University School of Medicine, Columbus, OH, USA

**Keywords:** intracranial hemorrhage, pediatric neurology, perinatal stroke

## Abstract

**Background:**

Perinatal stroke occurs in approximately 1 in 1100 live births. Large electronic health record (EHR) data can provide information on exposures associated with perinatal stroke in a larger number of patients than is achievable through traditional clinical studies. The objective of this study is to assess prevalence and odds ratios of known and theorized comorbidities with perinatal ischemic and hemorrhagic stroke.

**Methods:**

The data for patients aged 0-28 days with a diagnosis of either ischemic or hemorrhagic stroke were extracted from the Cerner Health Facts Electronic Medical Record (EMR) database. Incidence of birth demographics and perinatal complications were recorded. Odds ratios were calculated against a control group.

**Results:**

A total of 535 (63%) neonates were identified with ischemic stroke and 312 (37%) with hemorrhagic stroke. The most common exposures for ischemic stroke were sepsis (n = 82, 15.33%), hypoxic injury (n = 61, 11.4%), and prematurity (n = 49, 9.16%). The most common comorbidities for hemorrhagic stroke were prematurity (n = 81, 26%) and sepsis (n = 63, 20%). No perinatal ischemic stroke patients had diagnosis codes for cytomegalovirus disease. Procedure and diagnosis codes related to critical illness, including intubation and resuscitation, were prominent in both hemorrhagic (n = 46, 15%) and ischemic stroke (n = 45, 8%).

**Conclusion:**

This electronic health record–based study of perinatal stroke, the largest of its kind, demonstrated a wide variety of comorbid conditions with ischemic and hemorrhagic stroke. Sepsis, prematurity, and hypoxic injury are associated with perinatal hemorrhagic and ischemic stroke, though prevalence varies between types. Much of our data were similar to prior studies, which lends validity to the electronic health record database in studying perinatal stroke.

Perinatal stroke is an important cause of morbidity and mortality in pediatrics. Though there is no universal consensus about perinatal stroke definition, the American Heart Association's most recent scientific statement on the management of stroke in neonates defines it as occurring from 28 weeks’ gestation to 28 days postterm.^1^ After hypoxic-ischemic encephalopathy and intraventricular hemorrhage, stroke (with a rate estimated from about 1 in 1100 to 1 in 5000 live births), is the third most common neurologic injury neonates face.^2,3^ Besides the immediate life-threatening symptoms of encephalopathy and seizures, long-term effects can range from minimal symptoms to lifelong neurologic impairment with significant cognitive and functional motor defects.^4–6^ Perinatal ischemic stroke is the most common cause of hemiplegic cerebral palsy in term infants and the most common subtype of perinatal stroke.^7^

The pathophysiology of perinatal stroke is something of a “black box,” and there is an enormous amount that remains to be understood about this disease. The pathogenesis is complex and multifactorial, including placental complications, prothrombotic state, and physiological stressors associated with labor.^8,9^ Important steps have been made in recent decades to further characterize and study perinatal stroke, including work by the International Pediatric Stroke Study, national surveys from children's hospitals, regional and national registries, and prospective multicenter registries.^2,8–11^ Despite the work of these important studies, the exact pathophysiology of perinatal stroke remains elusive, and huge gaps remain in the understanding of risk factors and pathogenesis. Because of this gap in knowledge, widely applicable preventative, hyperacute, or acute strategies still do not exist.^1^

Large electronic health record (EHR) databases are well suited for conducting studies on rare diseases and outcomes given the large population of patients in the set. Such studies cannot typically be done in traditional clinical, case-control, or low-scale cohort studies. Electronic health record–based retrospective, observational studies allow researchers to collect and analyze this diverse information without the cost or time commitment of traditional studies. Consequently, we set out to use a large retrospective electronic health record database to study potential exposures associated with perinatal stroke. The objective of this study is to assess prevalence and odds ratios of known and theorized comorbidities with perinatal ischemic and hemorrhagic stroke. This study is observational, exploratory, and hypothesis-generating, serving to assess risk factors associated with perinatal stroke and assess the use of electronic health record data in the study of associations between birth-related complications and perinatal ischemic and hemorrhagic stroke.

## Subject and Methods

The data used for this project were extracted from the Cerner Health Facts EMR database (version 2018) per institutional review board approval.^12^ The Cerner Health Facts EMR database contains approximately 69 million patients, including 18.7 million patients aged 20 years or younger, across 750 hospitals and clinics nationwide. Pediatric patients were admitted or evaluated at 712 of these hospitals and clinics. This EMR data set contains data from 2000 to 2018, but because of a notable lack of patient data before 2009, for this study, we only examined patient records from 2009 to 2018.

### Selection of Stroke Patients

**Figure 1.**
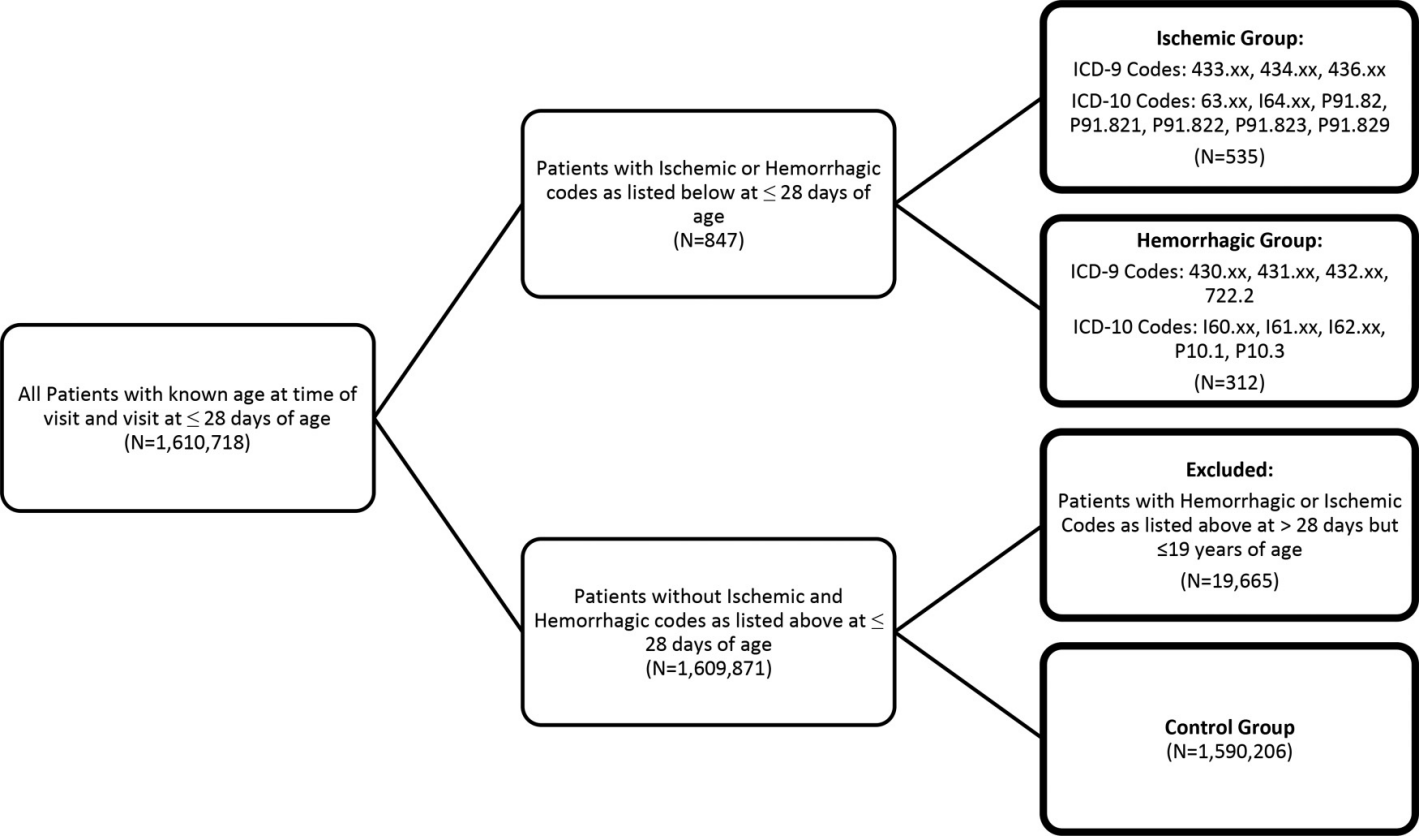
Flowchart of patient enrollment criteria.

Patients who were diagnosed with a stroke, as determined by ICD codes, at or before 28 days of age were extracted from the Cerner EMR database. The date of stroke was determined by the first presentation of a stroke-related ICD code, and all patients are only included once in the data set, regardless of the number of strokes they presented with. Patients with missing age were excluded. Patients were divided into ischemic and hemorrhagic stroke for all analyses. Ischemic stroke was defined as arterial ischemic stroke. Hemorrhagic stroke was defined as intracerebral hemorrhage and subarachnoid hemorrhage. Codes for cerebral venous sinus thrombosis, intraventricular hemorrhage, subdural hemorrhage, or epidural hemorrhage were not included in our search criteria. Patient enrollment is outlined in Figure 1.

### Selection of Control Subjects

Patients with a recorded visit at 28 days of age or earlier were extracted from the Cerner EMR database. Stroke patients, if they had a stroke, as defined above, in the Cerner system before or at 19 years of age, were excluded from the control group. All remaining neonates were included as the control group.

### Exposure Definition

Twenty-four exposures associated with birth were examined. Exposure definitions are not mutually exclusive, and an individual subject may have multiple exposures. Exposures may occur before, after, or concurrently with the diagnosis of stroke; however, only exposures occurring in the first 28 days of life are included. The receipt of aspirin was determined by a record of administration of any medication containing the word “aspirin.” The receipt of antiepileptic was determined by a record of administration of any medication containing the words “levetiracetam,” “Keppra,” “phenobarbital,” “phenytoin,” or “fosphenytoin.” Resuscitation or intensive care services was defined by receipt of procedure codes (CPT) for perinatal resuscitation (99465), intubation (31500), laryngoscopy (31515), umbilical catheterization (36510), or surfactant administration (94610), or by administration of a medication containing the word “epinephrine.” All other exposures were defined by *International Classification of Diseases, Ninth* (*ICD-9*) and *Tenth Revision* (*ICD-10*) codes, as specified in Appendix AA.

### Statistical Analysis

All analyses were performed using R (R Core Team, 2021). All analyses were stratified by stroke type—ischemic and hemorrhagic—with the nonstroke neonate controls as the comparison group. Exposures were summarized by count, proportion, unadjusted and adjusted odds ratios, and 95% confidence intervals for odds ratios. Continuous demographic characteristics were summarized using mean, standard deviation, median, and interquartile range. Categorical and binary demographic characteristics were summarized using count and proportion. Odds ratios are omitted where fewer than 5 neonates in either the stroke or control group had the given exposure. Statistical significance is determined through the use of the 95% confidence intervals for the odds ratio, where a covariate is considered not statistically significant if the 95% confidence interval includes 1 and considered statistically significant if the odds ratio does not include 1. This indicates a standard significance level of .05 as the cutoff for statistical significance.

Multivariable analysis was performed on a subset of comorbidities. Comorbidities were excluded from multivariable analysis if they had fewer than 5 neonates in the stroke or control group, had overlapping *ICD* codes with other comorbidities (extreme prematurity and extreme low birth weight), were at risk of cross-diagnosis with stroke (seizures), or could have occurred in response to the first stroke (aspirin and antiepileptic use). We present unadjusted odds ratios, a model for the adjusted odds of stroke from all nonexcluded comorbidities (full model), and the results of a multivariable model using a stepwise Akaike information criterion (AIC) variable selection method. Excluded comorbidities will be indicated in the corresponding table, along with reason for exclusion in the footnote.

## Results

From the Cerner EMR database, 312 neonates were identified with a diagnosis of hemorrhagic stroke, 535 neonates with a diagnosis of ischemic stroke, and 1,590,206 neonates did not have a diagnosis of stroke and were used as the control population. The incidence of ischemic stroke was 1 in 2974, and the incidence of hemorrhagic stroke was 1 in 5100 neonates. Neonates were, on average, older at the time of hemorrhagic stroke (6.42 ± 8.65 days) than at the time of ischemic stroke (4.90 ± 8.02 days) or at the time of recorded visit in the control population (3.33 ± 6.65 days). Hemorrhagic stroke patients were also more often male (57.67%) than patients in the ischemic (50.47%) or nonstroke neonate (51.58%) populations. Similarly, hemorrhagic stroke patients were also more likely to be African American (24.15%) than patients in the ischemic (13.32%) or control (16.85%) groups. Patients with ischemic stroke or hemorrhagic stroke were less likely to be Asian than those in the nonstroke neonate group (4.07%) with 1.69% and 1.36%, respectively. Although the highest percentage of hospital admissions were in 2014 for the control group, with 14.08%, the highest percentage of hospital admissions were in 2015 (22.76%) for hemorrhagic stroke neonates and 2013 (24.30%) for ischemic stroke neonates. Additional demographic data are presented in Table 1.

**Table 1.** Demographic Characteristics for Hemorrhagic, Ischemic, and Nonstroke Control Neonates.

	Hemorrhagic	Ischemic	Control
	(n = 312)	(n = 535)	(n = 1,590,206)
Age, d			
Median (IQR)	1.00 (0.00, 12.00)	0.00 (0.00, 6.00)	0.00 (0.00, 3.00)
Mean (STD)	6.42 (8.65)	4.90 (8.02)	3.33 (6.65)
**Gender**, n (%)			
Male	173 (57.67)	270 (50.47)	803,175 (51.58)
Female	127 (42.33)	253 (47.29)	753,952 (48.42)
Missing	12	12	33,103
**Race, n (%)**			
African American	71 (24.15)	63 (13.32)	241,125 (16.85)
Asian	4 (1.36)	8 (1.69)	58,278 (4.07)
Hispanic	8 (2.72)	16 (3.38)	47,458 (3.32)
White	175 (59.52)	331 (69.98)	896,616 (62.65)
Other	34 (11.56)	55 (11.63)	187,773 (13.12)
Missing	18	62	158,956
**Admission year, n (%)**			
2009	11 (3.53)	52 (9.72)	98,852 (6.22)
2010	14 (4.49)	38 (7.10)	120,602 (7.58)
2011	31 (9.94)	49 (9.16)	144,689 (9.10)
2012	36 (11.54)	73 (13.64)	164,045 (10.32)
2013	55 (17.63)	130 (24.30)	211,095 (13.27)
2014	69 (22.12)	121 (22.62)	223,902 (14.08)
2015	71 (22.76)	67 (12.52)	210,380 (13.23)
2016	7 (2.24)	4 (0.75)	191,389 (12.04)
2017	9 (2.88)	1 (0.19)	168,319 (10.58)
2018	9 (2.88)	0 (0.00)	56,933 (3.58)

**Figure 2.**
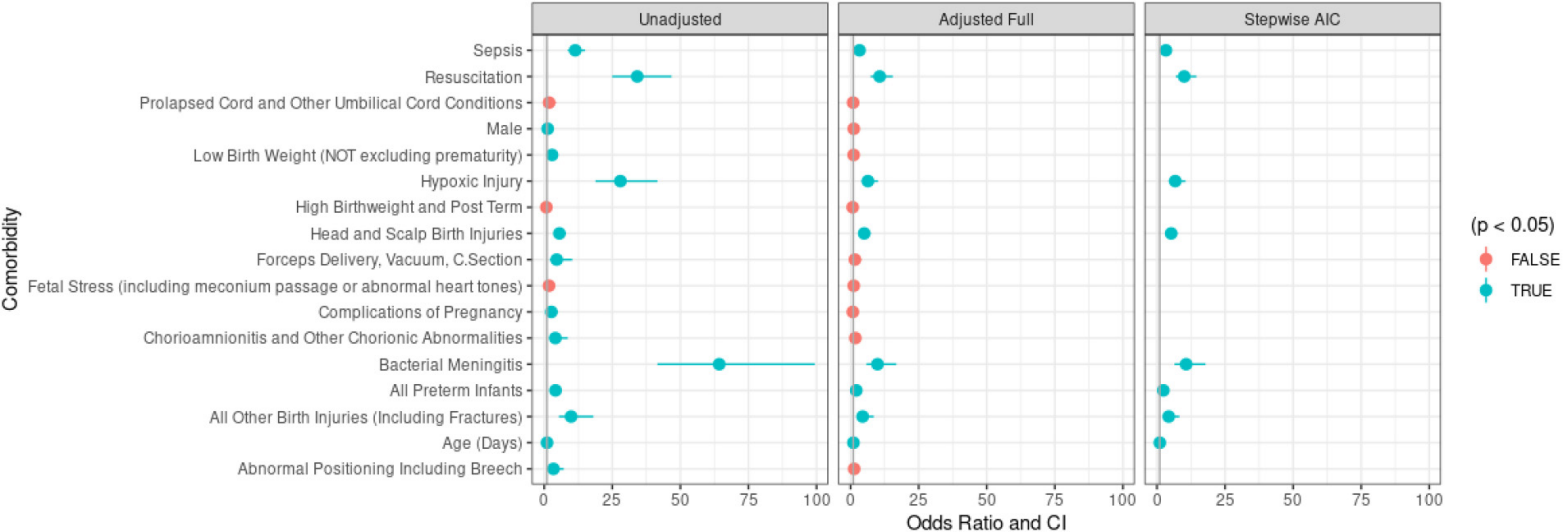
Hemorrhagic models.

Associated exposure proportions, unadjusted odds ratios, and adjusted odds ratios for perinatal hemorrhagic stroke are presented in Table 2 and Figure 2. Statistically significant results are represented in bold in the table and by color in the figure. The most prevalent exposure in the perinatal hemorrhagic stroke population is being born prematurely, present in 25.96% of hemorrhagic stroke patients (Table 2). Similarly, 18.91% of hemorrhagic stroke neonates had a low birth weight. Another common comorbidity in the hemorrhagic stroke population is sepsis, occurring in 63 (20.19%) neonates. Most of the examined exposures were statistically associated with hemorrhagic stroke in unadjusted models. Compared to the control population, bacterial meningitis and requiring resuscitation were the most strongly correlated with hemorrhagic stroke, with unadjusted OR = 64.26 (95% CI: 41.56, 99.36) and OR = 34.16 (95% CI: 24.96, 46.75), respectively. Hypoxic injury and extreme prematurity are also strongly associated with hemorrhagic stroke, having unadjusted ORs of 27.98 (95% CI: 18.84, 41.56) and 11.72 (95% CI: 7.95, 17.29), respectively.

**Table 2.** Prevalence and Odds Ratios With 95% Confidence Intervals for Perinatal Exposures in Hemorrhagic Stroke Patients.

	Hemorrhagic, n (%) (n = 312)	Control, n (%) (n = 1,590,206)	Unadjusted odds ratio (95% CI)	Adjusted odds ratio: full (95% CI)	Adjusted odds ratio: stepwise AIC (95% CI)
					
Age (d)			**1.05** **(** **1.04, 1.06)**	**1.05** (**1.04, 1.06)**	**1.05 (1.04,1.06)**
Male			**1.28** (**1.02, 1.61)**	1.19 (0.94, 1.50)	N/A
Abnormal positioning, including breech	7 (2.24)	10,675 (0.67)	**3.40** (**1.60, 7.19)**	1.33 (0.57, 3.10)	–^a^
All other birth injuries, including fractures	11 (3.53)	5860 (0.37)	**9.88** (**5.41, 18.04)**	**4.42** (**2.30, 8.51)**	**4.33 (2.28, 8.22)**
All preterm infants	81 (25.96)	123,714 (7.78)	**4.16** (**3.23, 5.35)**	**2.09** (**1.39, 3.12)**	**2.36 (1.77, 3.15)**
Bacterial meningitis	22 (7.1)	1875 (0.12)	**64.26** (**41.56, 99.36)**	**9.90** (**5.84, 16.79)**	**10.73 (6.46, 17.82)**
Chorioamnionitis and other chorionic abnormalities	7 (2.24)	8873 (0.56)	**4.09** (**1.93, 8.66)**	1.75 (0.80, 3.83)	–^a^
Complications of pregnancy	9 (2.88)	17,798 (1.12)	**2.62** (**1.35, 5.09)**	0.84 (0.40, 1.73)	–^a^
Cytomegaloviral disease^b^	2 (0.64)	391 (0.02)	N/A	N/A	N/A
Extreme low birth weight^c^	17 (5.44)	9419 (0.59)	**9.67** (**5.93, 15.78)**	N/A	N/A
Extreme prematurity^c^	28 (8.97)	13,266 (0.83)	**11.72** (**7.95, 17.29)**	N/A	N/A
Fetal stress, including meconium passage or abnormal heart tones	7 (2.24)	20,398 (1.28)	1.77 (0.83, 3.74)	1.17 (0.54, 2.53)	–^a^
Forceps delivery, vacuum, c-section	6 (1.92)	6762 (0.43)	**4.59** (**2.05, 10.30)**	1.59 (0.63, 3.97)	–^a^
Head and scalp birth injuries	33 (10.58)	33,004 (2.08)	**5.58** (**3.89, 8.01)**	**5.01** (**3.40, 7.37)**	**5.22 (3.60, 7.58)**
High birthweight and postterm	19 (6.09)	118,946 (7.48)	0.80 (0.50, 1.28)	0.78 (0.48, 1.27)	–^a^
Hypoxic injury	27 (8.65)	5366 (0.34)	**27.98** (**18.84, 41.56)**	**6.37** (**4.03, 10.06)**	**6.75 (4.30, 10.50)**
Low birth weight (*not* excluding prematurity)	59 (18.91)	118,888 (7.48)	**2.89** (**2.17, 3.83)**	1.14 (0.75, 1.72)	–^a^
Placental previa and other placental abnormalities^b^	3 (0.96)	2036 (0.13)	N/A	N/A	N/A
Preterm, >28 wk^c^	47 (15.06)	103,020 (6.48)	**2.56** (**1.88, 3.49)**	N/A	N/A
Prolapsed cord and other umbilical cord conditions	5 (1.60)	14,339 (0.90)	1.79 (0.74, 4.33)	0.94 (0.38, 2.33)	–^a^
Seizures^d^	81 (25.96)	6174 (0.39)	**89.96** (**69.76, 116.02)**	N/A	N/A
Sepsis	63 (20.19)	34,525 (2.17)	**11.40** (**8.65, 15.03)**	**3.28** (**2.29, 4.72)**	**3.30 (2.32, 4.70)**
Spinal cord, brachial plexus, and other nerve injuries^b^	2 (0.64)	1758 (0.11)	N/A	N/A	N/A
Neonate requiring resuscitation or intensive care (procedures and medications)	46 (14.74)	8010 (0.50)	**34.16** (**24.96, 46.75)**	**10.69** (**7.36, 15.52)**	**10.02 (6.92, 14.51)**
Aspirin in first 28 d^e^	5 (1.60)	1864 (0.12)	**13.88** (**5.73, 33.62)**	N/A	N/A
Antiepileptic before 28 d^e^	72 (23.08)	4606 (0.29)	**103.27** (**79.24, 134.60)**	N/A	N/A

Abbreviations: AIC, Akaike information criterion; N/A, not available.

^a^ Comorbidity was not selected by the AIC stepwise selection method.

^b^ Comorbidity excluded for fewer than 5 recorded occurrences.

^c^ Comorbidity excluded for overlap with other comorbidities.

^d^ Comorbidity excluded because of risk for misdiagnosis as stroke.

^e^ Comorbidity excluded because of being a potential result of stroke.

In the multivariable models, the adjusted odds ratios are all closer to the null. When adjusting for other covariates, low birth weight, forceps delivery, complications of pregnancy, chorionic abnormalities, and abnormal positioning are no longer statistically significant comorbidities for neonatal hemorrhagic stroke despite being significant in the univariate models. In the multivariable model, the adjusted odds ratios for requiring resuscitation (OR = 10.69, 95% CI: 7.36, 15.52), bacterial meningitis (OR = 9.90, 95% CI: 5.84, 16.79), and hypoxic injury (OR = 6.37, 95% CI: 4.03, 10.06) remain high. The stepwise AIC variable selection chose a model with age, all other birth injuries, bacterial meningitis, prematurity, head and scalp birth injuries, hypoxic injury, sepsis, and requiring resuscitation.

**Figure 3.**
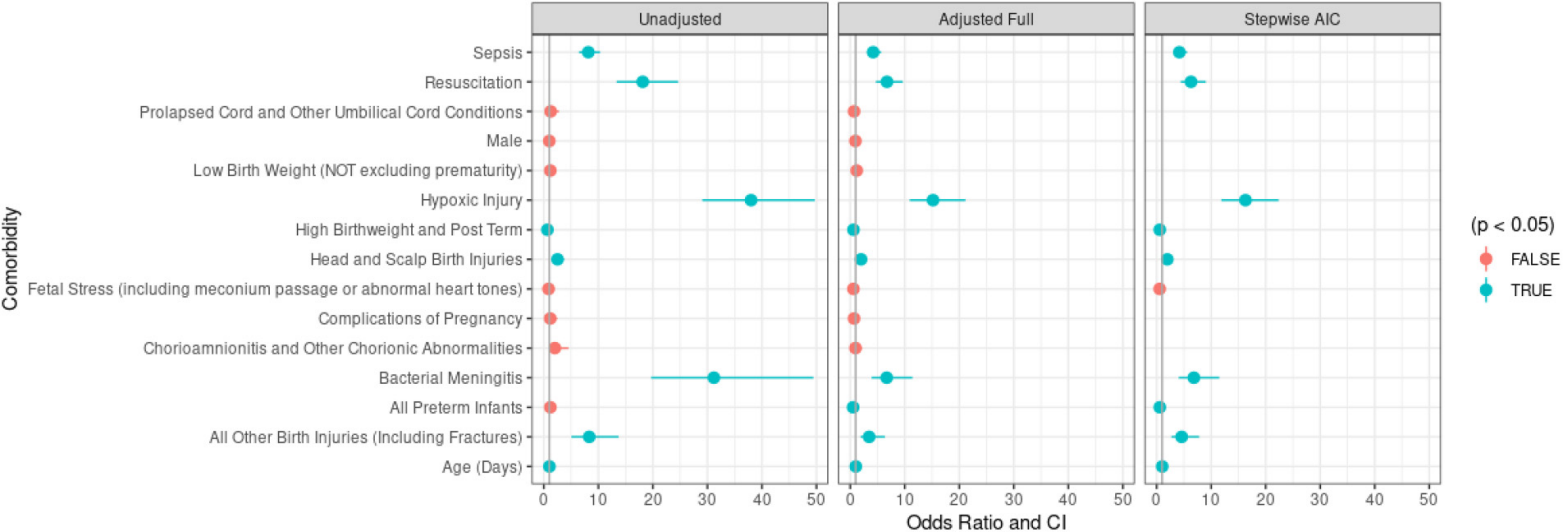
Ischemic models.

Prevalence values for ischemic stroke are generally lower than prevalence values for hemorrhagic stroke (Table 3). Sepsis and hypoxic injuries were the most commonly occurring exposures for perinatal ischemic stroke with 15.33% and 11.40% prevalence, respectively. Hypoxic injury and bacterial meningitis (3.6%) represented the largest unadjusted odds ratios, with OR = 38.01 (95% CI: 29.07, 49.69) and OR = 31.19 (95% CI: 19.69, 49.41), respectively. Other factors significantly associated with perinatal stroke include requiring resuscitation, which is present in 8.41% of ischemic stroke cases and has an unadjusted odds ratio of 18.14 (95% CI: 13.36, 24.64) and sepsis (15.33%) with an unadjusted odds ratio of 8.16 (95% CI: 6.45, 10.32), and all other birth injuries, including fractures (2.99%) with an odds ratio of 8.34 (95% CI: 5.06, 13.72). High birth weight and post-term birth occurred in 5.05% of ischemic stroke patients and was associated with a reduced risk of ischemic stroke in neonates as compared to nonstroke neonates, with an unadjusted odds ratio of 0.66 (95% CI: 0.45, 0.97). We also investigated neonates that had received aspirin. Eighty-eight (16.45%) of neonates received aspirin in the first 28 days of life, for an unadjusted odds ratio of 167.75 (95% CI: 132.88, 211.78) when compared to neonates without stroke. Additional prevalences for perinatal ischemic stroke is presented in Table 3 and Figure 3. Statistically significant results are represented in bold in the table and by color in the figure.

**Table 3.** Prevalence and Odds Ratios with 95% Confidence Intervals for Perinatal Exposures in Ischemic Stroke Patients.

	Ischemic, n (%) (n = 535)	Control, n (%) (n = 1,590,206)	Unadjusted odds ratio (95% CI)	Adjusted odds ratio: full (95% CI)	Adjusted odds ratio: stepwise AIC (95% CI)
					
Age (d)			**1.03** **(** **1.02, 1.05)**	**1.02** (**1.01, 1.04)**	**1.03 (1.02, 1.04)**
Male			1.00 (0.84, 1.19)	0.96 (0.81, 1.14)	N/A
Abnormal positioning, including breech^a^	1 (0.19)	10,675 (0.67)	N/A	N/A	N/A
All other birth injuries, including fractures	16 (2.99)	5860 (0.37)	**8.34** (**5.06, 13.72)**	**3.47** (**1.89, 6.36)**	**4.59 (2.70, 7.80)**
All preterm infants	49 (9.16)	123,714 (7.78)	1.20 (0.89, 1.60)	**0.53** (**0.35, 0.81)**	**0.58 (0.42, 0.80)**
Bacterial meningitis	19 (3.6)	1875 (0.12)	**31.19** (**19.69, 49.41)**	**6.69** (**3.94, 11.41)**	**6.83 (4.06, 11.49)**
Chorioamnionitis and other chorionic abnormalities	6 (1.12)	8873 (0.56)	2.02 (0.90, 4.52)	0.97 (0.42, 2.21)	^b^
Complications of pregnancy	7 (1.31)	17,798 (1.12)	1.17 (0.56, 2.47)	0.71 (0.33, 1.52)	^b^
Cytomegaloviral disease^a^	0 (0.00)	391 (0.02)	N/A	N/A	N/A
Extreme low birth weight^c^	7 (1.31)	9419 (0.59)	**2.23** (**1.06, 4.69)**	N/A	N/A
Extreme prematurity^c^	7 (1.31)	13,266 (0.83)	1.58 (0.75, 3.32)	N/A	N/A
Fetal stress, including meconium passage or abnormal heart tones	6 (1.12)	20,398 (1.28)	0.87 (0.39, 1.95)	0.59 (0.26, 1.33)	0.56 (0.25, 1.26)
Forceps delivery, vacuum, c-section^a^	2 (0.37)	6762 (0.43)	N/A	N/A	N/A
Head and scalp birth injuries	27 (5.05)	33,004 (2.08)	**2.51** (**1.70, 3.69)**	**1.99** (**1.33, 2.98)**	**1.96 (1.31, 2.92)**
High birthweight and postterm	27 (5.05)	118,946 (7.48)	**0.66** (**0.45, 0.97)**	**0.60** (**0.40, 0.89)**	**0.57 (0.39, 0.85)**
Hypoxic injury	61 (11.40)	5366 (0.34)	**38.01** (**29.07, 49.69)**	**15.18** (**10.91, 21.13)**	**16.29 (11.86, 22.37)**
Low birth weight (*not* excluding prematurity)	47 (8.79)	118,888 (7.48)	1.19 (0.88, 1.61)	1.19 (0.80, 1.79)	^b^
Placental previa and other placental abnormalities^a^	3 (0.56)	2036 (0.13)	N/A	N/A	N/A
Preterm, greater than 28 wk gestation^c^	35 (6.54)	103,020 (6.48)	1.01 (0.72, 1.42)	N/A	N/A
Prolapsed cord and other umbilical cord conditions	6 (1.12)	14,339 (0.90)	1.25 (0.56, 2.79)	0.71 (0.31, 1.62)	^b^
Seizures^d^	187 (34.95)	6174 (0.39)	**137.87** (**115.22, 164.97)**	N/A	N/A
Sepsis	82 (15.33)	34,525 (2.17)	**8.16** (**6.45, 10.32)**	**4.21** (**3.12, 5.68)**	**4.17 (3.11, 5.58)**
Spinal cord, brachial plexus, and other nerve injuries^a^	1 (0.19)	1758 (0.11)	N/A	N/A	N/A
Neonate requiring resuscitation or intensive care (procedures and medications)	45 (8.41)	8010 (0.50)	**18.14** (**13.36, 24.64)**	**6.74** (**4.71, 9.64)**	**6.31 (4.41, 9.01)**
Aspirin in the first 28 d^e^	88 (16.45)	1864 (0.12)	**167.75** (**132.88, 211.78)**	N/A	N/A
Antiepileptic in the first 28 d^e^	146 (27.29)	4606 (0.29)	**129.20** (**106.59, 156.62)**	N/A	N/A

Abbreviations: AIC, Akaike information criterion; N/A, not available.

^a^ Comorbidity excluded for fewer than 5 recorded occurrences.

^b^ Comorbidity was not selected by the AIC stepwise selection method.

^c^ Comorbidity excluded for overlap with other comorbidities.

^d^ Comorbidity excluded due to risk for misdiagnosis as stroke.

^e^ Comorbidity excluded due to being a potential result of stroke.

In the multivariable models, no comorbidities that were statistically significant in the univariate models lose their significance when other comorbidities are adjusted for; however, the magnitude of association changes for some comorbidities. Notably, the adjusted odds ratios for hypoxic injury (OR = 15.18. 95% CI: 10.91, 21.13), requiring resuscitation (OR = 6.74, 95% CI: 4.71, 9.64), bacterial meningitis (OR = 6.69, 95% CI: 3.94, 11.41), and sepsis (OR = 4.21, 95% CI: 3.12, 5.68), remain high and high birth weight and postterm birth remains protective (OR = 0.66, 95% CI: 0.45, 0.97). Preterm birth has a negative association with arterial ischemic stroke in the multivariable model. The stepwise AIC variable selection chose a model with age, all other birth injuries, bacterial meningitis, prematurity, fetal stress (not statistically significant), high birth weight and postterm birth, head and scalp birth injuries, hypoxic injury, sepsis, and requiring resuscitation.

## Discussion

In this population-based study of electronic health record data in perinatal stroke, we analyzed the association of potential risk factors with perinatal ischemic stroke and hemorrhagic stroke. This study analyzed more than 500 patients with ischemic stroke and more than 300 with hemorrhagic stroke, the largest of its kind in perinatal stroke. This study was racially diverse and took data from both academic and nonacademic centers. Much of our data were consistent with data from prior single-center and multicenter academic hospital-based investigations, including high frequency of seizures and the tendency of patients with perinatal stroke to have systemic illness and a difficult transition to extrauterine life.^10^ We also estimate an incidence of approximately 1 in 3000 neonates with ischemic stroke and 1 in 5000 with hemorrhagic stroke, which is similar to data from the Alberta Perinatal Stroke Project, a validated prospective study on perinatal stroke.^13^ These similar findings support the validity of electronic health record data as an information source for perinatal stroke for this article.

Though much of our work is consistent with prior data from the International Pediatric Stroke Study, Calgary Perinatal Stroke Project, and others, there are important differences in findings between this study and previous studies on perinatal stroke. We found a statistically significant association between head and scalp birth injuries (such as cephalohematoma) and diagnosis codes related to trauma, such as long bone fractures, with both hemorrhagic and ischemic stroke. In the multivariable models, these associations were weaker, but remained statistically significant. We believe that this association should be interpreted cautiously. “Trauma,” whether through forceps extraction or other assisted delivery, has drawn significant interest as a risk factor for pediatric stroke from both parents and clinicians.^14^ Arterial stroke and intracranial hemorrhage in neonates has been theorized to be a result of direct trauma to large vascular structures, or stretching of arteries from the forces of labor.^9^ Prolonged labor, as well, has been reported to be a risk factor for perinatal stroke.^15^ However, to our knowledge, there has only been 1 documented case of perinatal infarction attributable to dissection.^16^ Data from the International Pediatric Stroke Study found increased odds of worsened outcome in perinatal stroke patients delivered by vaginal delivery as compared to cesarean section, but “trauma” was recorded in less than 1% of patients in their cohort.^10^ A 2019 review article of perinatal stroke by Dunbar and Kirton concluded that prior associations of mechanical factors associated with birth and ischemic or hemorrhagic stroke, such as restrictive birth canal or forceps-assisted delivery, have not demonstrated causality.^17^ Additionally, a recent study of perinatal hemorrhagic stroke, which defined trauma as skull fracture or major soft tissue bruising, found evidence of trauma in only 4 of 51 perinatal hemorrhagic stroke patients, with the authors concluding that trauma is rarely associated with perinatal intracranial hemorrhage.^2^ As “trauma” ICD codes decreased in statistical significance in our multivariable model, it is possible that the association of “trauma” ICD codes and neonatal stroke found in this study is confounded by other factors or comorbidities, such as perinatal asphyxia or difficult delivery. Perinatal stroke remains poorly understood, but an association between a difficult transition to extrauterine life remains a well-established and repeatedly reported risk factor.^10,18,19^ Consequently, we believe that any associations between traumatic injuries at birth and stroke should be interpreted cautiously, and this study, based on its retrospective nature and electronic health record design, does not demonstrate causality between traumatic skull and bone injuries and perinatal stroke.

Additionally, in our assessment of the effect of birth-associated exposures on the risk of ischemic stroke, we did not find any statistically significant association with chorioamnionitis, fetal stress (such as meconium passage), low birth weight, or prolapsed cord, despite these factors being well-documented risk factors in perinatal stroke.^13,17^ We also found no association between congenital cytomegalovirus infection, though there have been prior data supporting an association between cytomegalovirus and neonatal stroke.^20^ Prematurity had a very mild protective effect, but only in our multivariable model. In addressing the validity of electronic health record data to study perinatal stroke, we found that ICD codes for many conditions, such as chorioamnionitis and prolapsed cord, were reported infrequently in the control population, and thus were likely underreported in the stroke groups as well. It is likely that the rate of the chorioamnionitis, fetal stress, prematurity, low birth weight, and prolapsed cord were greatly underestimated in this study because of the nature of this electronic health record data. Consequently, we believe any association (or lack thereof) regarding maternal risk factors for perinatal stroke should be interpreted with caution in electronic health record studies of perinatal stroke if the infants cannot be linked to their mother's chart, as was the case in our study.

There are several other limitations with this study, which are shared among large electronic health record studies in general.^21^ Because we are reliant on diagnosis codes for our stroke population, we were unable to verify that all cases were truly stroke, as we cannot assess the imaging and clinical history of the patients in the study. Some patients coded as ischemic stroke may have been more consistent with hypoxic injury or periventricular infarction. Similarly, some patients coded as hemorrhagic stroke in fact may have had preterm intraventricular hemorrhage. However, a recent validation study in the perinatal period found a strong correlation between *ICD-10* diagnosis and arterial ischemic stroke in the pediatric population.^22^ Because of the retrospective aspects of this electronic health record study, we also cannot establish temporality of the comorbidities. All results presented in this article are exploratory. Future work in this field could look to leverage electronic health record data to find novel or unexpected associations of *ICD* codes with perinatal stroke.

## Conclusion

In this large study of electronic health records, we assessed the agreement between electronic health record data on perinatal stroke and prior studies. We found an incidence of perinatal ischemic stroke and perinatal hemorrhagic stroke of approximately 1 in 3000 and 1 in 5000, respectively. We found a tendency for patients with perinatal stroke to have medical illness, including a propensity for sepsis, meningitis, and hypoxic-ischemic encephalopathy. This is the largest cohort of perinatal stroke patients analyzed, in particular with regard to neonatal hemorrhagic stroke. The agreement of our data with prior studies supports the accuracy of electronic health record data for the study of perinatal stroke, though we found limitations in the ability to analyze antenatal and maternal risk factors with perinatal stroke. Overall, this study provides important complementary information about perinatal ischemic and hemorrhagic stroke and suggests that risk factors analyzed in prior data sets are likely generalizable to a larger, nationwide population of neonates.

## Appendix A

*ICD* Codes Used for Data Extraction

**Table:**

Comorbidity	*ICD-9* Codes	*ICD-10* Codes
Complications of pregnancy	761.xx	P01.xx
Placenta previa and other placental abnormalities	762.0, 762.1, 762.2, 762.3	P02.0, P02.1, P02.2, P02.3
Prolapsed cord and other umbilical cord conditions	762.4, 762.5, 762.6	P02.4, P02.5, P02.6
Chorioamnionitis and other chorionic abnormalities	762.7, 762.8, 762.9	P02.7 P02.8, P02.9
Abnormal positioning, including breech	763.0, 763.1	P03.0, P03.1
Forceps delivery, vacuum, or c-section	763.2, 763.3, 763.4	P03.2, P03.3, P03.4
Fetal stress, including meconium passage or abnormal heart tones	763.8	P03.8
Low birth weight (not excluding prematurity), >1000 grams	764.00, 764.04-09, 764.10, 764.14-19, 764.20, 764.24-764.29, 764.90, 764.94-99, 765.04-09, 765.14-19	P05.04-09, P05.14-19, P07.1
Extreme low birth weight, <1000 grams	764.01-.03, 764.11-13, 764.21-23, 764.91-93, 765.01-03, 765.11-13	P05.01-03, P05.11-13, P07.0x
All preterm infants	765.0, 765.1, 765.20-28.	P07.2, P07.3
Extreme prematurity	765.0x, 765.21-24	P07.2
High birthweight and post-term	766.xx	P08.xx
Head and scalp birth injuries	767.1, 767.11, 767.19	P12.xx
Spinal cord, brachial plexus, and other nerve injuries	767.4, 767.5, 767.6, 767.7	P11.xx, P14.xx
All other birth injuries, including fractures	767.2, 767.3, 767.8, 767.9	P13.xx, P15.xx
Hypoxic injury	768.xx	P91.xx
Seizures	345.9, 780.39, 779.0, 345.xx	G40.xx, P90.xx
Sepsis	038.xx, 790.7, 995.92, 771.81, 112.83, 114.2, 115.01, 115.11, 115.91, 130.0, 136.2, 139.0, 321.xx, 323.xx, 047.xx, 048.xx, 049.xx, 053.0, 053.1, 054.72, 056.01, 062.xx, 063.xx, 064.xx, 066.2, 072.1, 072.2	R78.81, A40.xx, A41.xx, A42.7, A22.7, B37.7, A26.7, A28.2, A54.86, B00.7, A32.7, A24.1, A39.2, A39.3, A39.4, A20.7, A21.7, A48.3, P36.xx
Bacterial meningitis	036.xx, 013.xx, 003.21, 322.xx, 320.xx, 100.81, 098.82, 094.81, 094.2, 091.81, 090.42	G00.xx-G03.xx
Cytomegaloviral disease	771.1, 078.5, 484.1	B25.xx, P35.1, B27.1

Key: zz.xx indicates all codes from zz.00 to zz.99.
